# Efficacy and safety of Descemet’s membrane endothelial keratoplasty versus Descemet’s stripping endothelial keratoplasty: A systematic review and meta-analysis

**DOI:** 10.1371/journal.pone.0182275

**Published:** 2017-12-18

**Authors:** Saiqun Li, Liangping Liu, Wei Wang, Ting Huang, Xingwu Zhong, Jin Yuan, Lingyi Liang

**Affiliations:** 1 State Key Laboratory of Ophthalmology, Zhongshan Ophthalmic Center, Sun Yat-sen University, Guangzhou, China; 2 Hainan Eye Hospital, Zhongshan Ophthalmic Center, Sun Yat-sen University, Haikou, China; Xiamen University, CHINA

## Abstract

**Purpose:**

Based on current evidence, the efficiency and safety of Descemet’s membrane endothelial keratoplasty (DMEK) was compared with that of Descemet’s stripping endothelial keratoplasty (DSEK).

**Methods:**

Pubmed, Embase, Web of Science, the Cochrane Database and conference abstracts were comprehensively searched for studies that compared the efficacy and safety of DMEK and DSEK. The efficacy outcome was the postoperative best-corrected visual acuity (BCVA). The safety outcomes included the postoperative endothelial cell density (ECD) and complications such as graft detachment, graft rejection, graft failure, postoperative elevated intraocular pressure (IOP), tissue loss, etc. The outcomes were pooled using random-effects models with Stata 13.0 software. Heterogeneity was qualified with Q statistic and *I*^*2*^*/H*^*2*^ statistic. Publication bias was assessed using funnel plot, Begg rank correlation test, and Egger or Horbard linear regression.

**Results:**

19 articles were eligible, and 1124 eyes and 1254 eyes were included in the DMEK and DSEK groups, respectively. The overall pooled estimates showed a significantly better postoperative BCVA, a comparable ECD and an increased graft detachment rate in the DMEK group compared with the DSEK group (BCVA: mean difference (MD) = -0.15, 95% CI = -0.19 to -0.11, P<0.001; ECD: MD = 14.88, 95% CI = -181.50 to 211.27, P = 0.882; graft detachment rate: OR = 4.56, 95% CI = 2.43 to 8.58, P<0.001). Except for the postoperative ECD, which was changed to be higher in the DSEK group than the DMEK group, the learning curve did not have a marked effect on the comparison outcome of the BCVA and graft detachment rate based on the estimates pooled from studies that collected data during the DMEK learning phase (ECD (learning curve): MD = -361.24, 95% CI = -649.42 to -73.07, P = 0.014).

**Conclusion:**

Although DMEK is a more technically difficult and challenging procedure, it may represent a safe and more efficient alternative to DSEK for the treatment of corneal endothelial diseases, even during its learning curve.

## Introduction

In 2004, corneal transplantation took a great advance by introducing a new technique termed “Descemet’s stripping endothelial keratoplasty” (DSEK)[1–3]. In this new keratoplasty procedure, the patient’s diseased endothelium and Descemet’s membrane are replaced with posterior corneal stroma, Descemet’s membrane and endothelium from the donor cornea[1, 2, 4]. Since then, DSEK has been rapidly adopted by surgeons worldwide, and it has gradually become the standard surgical treatment for corneal endothelial problems, such as Fuchs corneal dystrophy, pseudophakic bullous keratopathy (PBK) and iridocorneal endothelial (ICE) syndrome, because of its short learning curve, good clinical outcomes, easier donor preparation and manipulation, and reproducible results[5, 6].

However, the interface opacification, optical irregularities, hyperopic refractive shift and thicker cornea caused by the extra stromal layers transplanted during the DSEK procedure may have a negative impact on postoperative visual quality[7, 8]. Therefore, to fully retain the anatomy of the recipient’s cornea, endothelial keratoplasty (EK) was used with the introduction by Melles in 2006 of “Descemet’s membrane endothelial keratoplasty” (DMEK), which transplants a lamella of Descemet’s membrane and endothelium without an adherent donor corneal stroma[9]. Since its introduction, the number of DMEK cases performed each year in the United States has doubled every year; however, DSEK is still the dominant surgical treatment of choice for endothelial diseases[10].

DMEK and DSEK both have advantages and disadvantages. Numerous studies have demonstrated that DMEK results in faster visual rehabilitation and better final visual acuity than DSEK[11–13]. However, despite the favorable visual outcomes achieved by DMEK, the technical challenges and steep technical learning curve appear to prevent many surgeons from abandoning DSEK in favor of DMEK[5, 14, 15]. Because of the thinner graft used in DMEK (e.g., 15 μm in DMEK versus 50–150 μm or thicker in DSEK), unfolding the DMEK graft in the anterior chamber can be more challenging. In addition, a higher graft detachment rate after DMEK might require more postoperative rebubbling or repositioning[6]. Therefore, the extra intraoperative and postoperative anterior chamber manipulation required during the DMEK procedure, as well as the difficulties in DMEK graft preparation, theoretically result in greater endothelial cell loss (ECL) in DMEK; however, although this outcome has been observed in only a few studies[7, 8], it has not been observed in many others[13, 16].

Until recently, a few comparative studies have reported the differences in efficiency and safety between DSEK and DMEK, and of the reported studies, many were small case series that presented inconsistent results, which limited the ability to draw definitive conclusions to better guide clinical practice[11–13, 16–22]. To the best of our knowledge, comparisons of the efficiency and safety between DSEK and DMEK have not been systematically reviewed and published. Therefore, we systematically analyzed the available literature to evaluate the efficiency and safety of DMEK versus DSEK.

## Materials and methods

This systematic review was performed in accordance with the PRISMA (Preferred reporting items for systematic reviewers and meta-analysis) statement issued by the Consolidated Standards of Reporting Trials (CONSORT) group (www.consort-statement.org)[23, 24]. No review protocol had been published.

### Literature search

Two reviewers systematically searched Pubmed, Embase, Web of Science, the Cochrane Database and conferences abstracts presented at the Association for Research and Vision in Ophthalmology (ARVO) (http://www.iovs.org/) for relevant articles from the databases’ inception to Jan 2017. The search terms included “Descemet’s membrane endothelial keratoplasty”, “Descemet’s stripping automated endothelial keratoplasty”, “Descemet’s stripping endothelial keratoplasty”, “DMEK”, “DSAEK” and “DSEK”. In addition, the bibliographies of the identified articles and reviews were manually checked to identify further publications. Restrictions were not placed on the study design or language in the literature search, and the studies were limited to human subjects.

### Inclusion and exclusion criteria

Two reviewers independently screened both the titles and abstracts from all identified articles. The full-text articles were retrieved for the articles that potentially matched the inclusion criteria. The inclusion criteria for eligibility were as follows: (1) comparisons of the efficacy and/or safety between DMEK and DSEK were reported; (2) prospective and retrospective comparative controlled clinical studies were performed, which were included because of the paucity of randomized controlled trials (RCTs) on DMEK and DSEK; and (3) the inclusion of at least one of the outcomes of interest. The inclusion of duplicated data that might lead to an overestimation of intervention effects was carefully avoided. Editorials, reviews, and letters to the editor were excluded. Studies in which the surgical techniques could not be defined and the outcome of comparison between two surgical techniques was not reported or could not be calculated were also excluded. Disagreements between the two reviewers were resolved by consensus or consultation with the senior authors.

### Data extraction

Two reviewers were involved in extracting the following data: (1) study characteristics, including the first author, year of publication, journal, country, study design, number of patients, patient demographics, indications for surgery and follow-up time; (2) efficacy outcomes, including postoperative BCVA; (3) safety outcomes, including postoperative ECD, and the incidence of complications, such as graft detachment, graft rejection, primary graft failure, graft loss during preparation, postoperative high intraocular pressure (IOP), etc. Discordant data were clarified, and missing data were obtained by contacting the study authors.

### Quality assessment

The level of evidence provided by the studies was graded in accordance with the Oxford Center for Evidence-Based Medicine (CEBM) scheme[25]. A modified version of the Newcastle-Ottawa Scale (NOS) was used to assess the methodological quality of nonrandomized studies[26]. The NOS consists of eight items grouped into three categories: selection, comparability and outcome. Studies were awarded a maximum of one star for each item within the selection and outcome categories and a maximum of two stars for comparability. The studies that were assigned a score >6 were considered of relatively high quality. Two reviewers independently evaluated the methodological quality of the included studies. A third senior reviewer was involved in the discussion and consensus to resolve disagreements on the quality assessment.

### Statistical analysis

The meta-analysis was performed using Stata 13.0 (StataCorp, College Station, Texas, USA). The continuous outcomes were presented as mean differences (MDs) with 95% confidence intervals (CIs), whereas the dichotomous outcomes were presented as odds ratios (ORs) or risk differences (RDs) with 95% CIs. Standard mean differences (SMDs) were not needed, if all results were reported in identical scales. In studies that reported continuous data as median and range values, the mean and standard deviation (SD) were estimated using the method proposed by Hozo et al.[27]. Heterogeneity among the studies was qualified with the Q statistic as well as *I*^*2*^/*H*^*2*^ statistic.

Heterogeneity is the norm and is often undetected [28]. Moreover, *I*^*2*^ was found to have low statistical power and wider confidence intervals with small numbers of studies like our study [29]. Thus, heterogeneity might be unidentified based on *I*^*2*^ statistic. In addition, fixed-effects model does not perform well only if there are very little between-study variations. For these reasons, random-effects model was used in all effect estimate pooling. As for weighting effects, Inverse Variance (IV) approaches was used for both continuous and dichotomous outcomes. There are numerous random-effects methods, including DerSimonian-Laird (D-L) family methods (standard D-L, bootstrap D-L) and other alternatives (simple Maximum Likelihood and Profile likelihood). Principally, standard D-L methods were used in the study. However, analysis using other more advanced methods than standard D-L methods, such as bootstrap D-L and Profile Likelihood methods, were also performed (data in S1 and S2 Tables).

Publication bias analysis and sensitivity analysis was performed only for BCVA, ECD and graft detachment rate. The publication bias was graphically checked by contour-enhanced funnel plot combined with “trill and fill” method. In addition, the publication bias was further assessed mathematically using the Begg rank correlation test, and Egger or Horbard linear regression. Horbard regression was only used for dichotomous outcomes. The “Proteus” phenomenon on the study outcomes over the years was assessed by cumulative meta-analysis. A P value of <0.05 was considered statistically significant.

## Results

### Literature search

**Fig 1:**
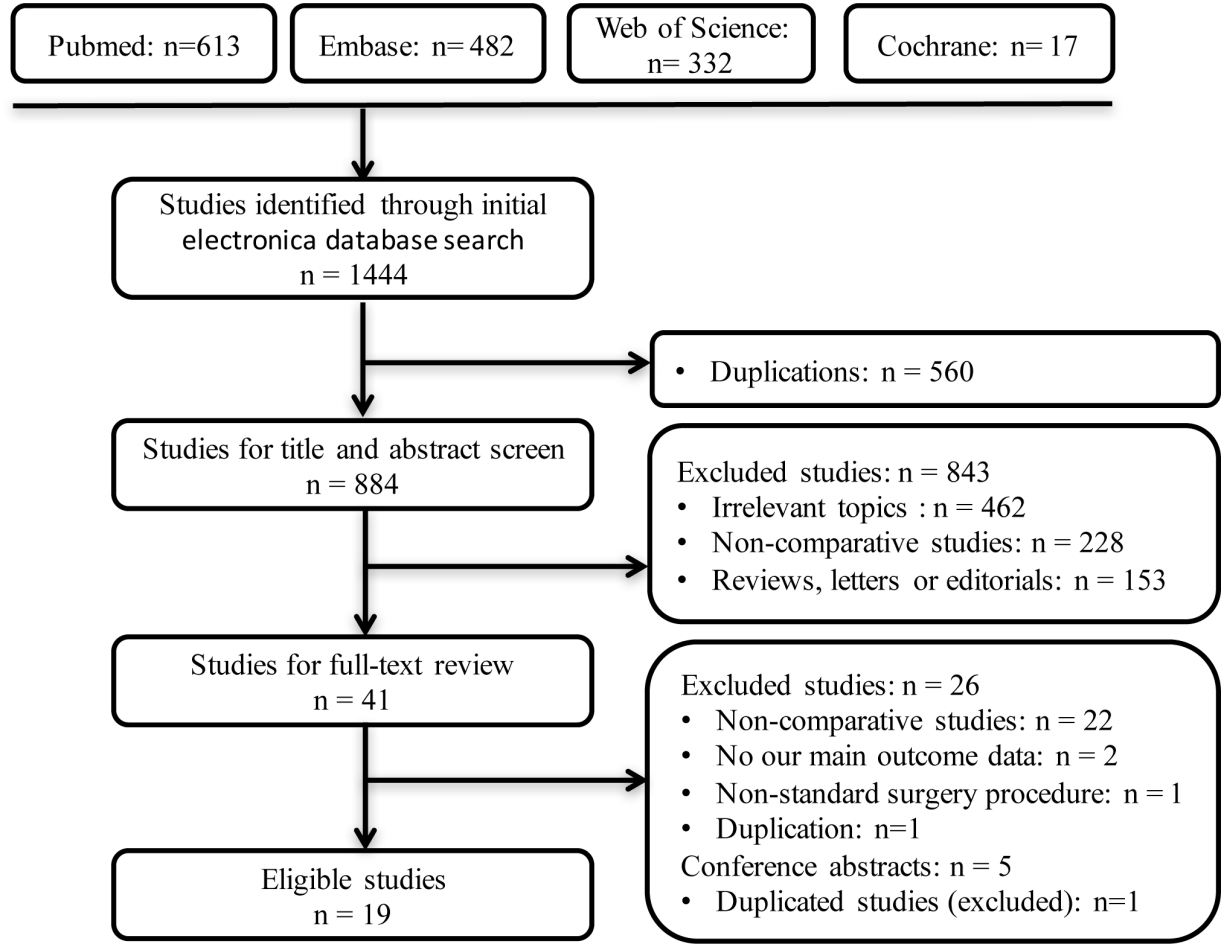
Flow chart of study identification.

The literature search process is shown in Fig 1. Our initial electronic database search yielded 884 non-duplicative individual articles. A total of 843 studies did not meet the inclusion criteria after the titles and abstracts were screened. The remaining 41 studies were retrieved for a full-text review. In all, 25 studies that did not use a probable control or report the outcomes of interest were further excluded. One additional study was excluded because of the use of hybrid techniques in the DMEK instead of a standard DMEK. A gray literature search revealed 5 conference abstracts presented at ARVO that were not published elsewhere, and one was excluded because it was later published as a full-text article that had already been included. Cross-referenced bibliographies did not yield additional eligible studies. Finally, 19 studies were included in this systematic review and meta-analysis.

### Study characteristics and quality assessment

Study and quality characteristics of the 19 identified articles are summarized in Table 1[11–13, 16–22, 30–40]. The eligible articles and abstracts included 15 retrospective control studies and 1 prospective non-randomized case series, and the study design of the remaining 3 meeting abstracts was not clarified. Two retrospective studies included prospective data collection. A total of 2278 patients with 2378 eyes were included in these studies, with 1124 eyes receiving DMEK and 1254 eyes receiving DSEK (including ultra-thin DSEK). A total of 82 patients from 5 studies underwent DMEK in one eye and DSEK in the other eye. The average follow-up period varied from 3.1 months to 22.55 months. The main indications for EK included Fuchs’ dystrophy, followed by PBK, ICE syndrome and regrafting. Most studies excluded patients with ocular comorbidities (e.g., glaucoma filtration surgeries, anterior chamber intraocular lens, aphakia, glaucoma tube, scleral fixed intraocular lenses), except one study that evaluated EK in eyes with an anterior chamber intraocular lens. The corneal transplantation with concurrent phacoemulsification and intraocular lens implantation (triple procedure) was performed if a cataract was observed at the time of surgery. A total of 12 studies stated that the donor graft for DSEK was prepared with microkeratome system equipment with a 300–400 μm head, 2 studies used DSEK grafts that were prepared manually, and 4 studies used precut DSEK grafts prepared by the eye bank. Overall, 10 studies reported preoperative or postoperative DSEK graft thicknesses and 5 studies used ultra-thin-DSEK grafts with an average grafts thickness of less than 120 μm. Most studies presented appropriate matchings between groups with respect to the patient demographics, surgical indications and donor characteristics. For quality assessment via NOS, 10 out of 19 studies had scores over 6, with the lowest at 4 (Table 1).

**Table 1:** Study characterises and quality assessment.

Study	Year	Country	Study Design	Indications	No. of eyes		Mean Age (years)		Follow-up time (months)		Quality score
					DMEK	DSEK	DMEK	DSEK	DMEK	DSEK	
Anshu	2012	USA	R	FED, PBK, regraft, ICE	141	598	66±11	66±12	13(3–40)	11(3–42)	6
Bhandari	2015	IN	R	FED	30	30	55.12±9.2	55.12±9.2	12	12	6
Droutsas	2016	GER	R	FED	25	25	71(44–89)	72(58–85)	12	12	6
Goldich	2014	CA	R	FED	10	10	72.5±13.5	72.5±13.5	9.6±2.2	36.5±15.4	4
Goldich	2015	CA	R	FED	17	17	72.6±11.3	72.6±11.3	At least 6	At least 6	5
Green^#^	2015	UK	R	FED	14	12	66(49–80)	68(26–86)	3.1(0.3–7.3)	4.1(0.3–8.9)	7
Guerra	2011	USA	R	FED	15	15	67(53–83)	67(53–83)	12	12	6
Hamzaoglu^#^	2015	POR	R	FED	100	100	70.6±9.6	68.1±11.0	6	6	6
Heinzelmann	2016	GER	R	FED, PBK	450	89	69±12	70±9	6.5±3.2	21.0±10.5	6
Liarakos	2013	GR	R	PBK(with AC-IOL)	14	7	62.4±20.7	72.4±12.6	At least 6	At least 6	4
Maier	2015	GER	R	FED	10	10	71±6	71±6	6.5±3.2	21.0±10.5	5
Philips^#^	2016	USA	R	FED	100	100	65.9	68.9	6	6	6
Rose-Nussbaumer^#^	2016	USA	P	FED	42	18	69±9.8	68±6.5	6	6	7
Rudolph	2012	GER	R	FED	30	20	68.77±9.54	69.70±10.17	6.50±1.20	22.55±11.80	5
Tourtas	2012	GER	R	FED, PBK	38	35	68.3±9	68.1±11	6	6	6
**ARVO Abstract**											
Yan^#^	2013	CA	R	FED, PBK	10	10/10 *	67.5	76/ 69*	6	6	4
Davis-Boozer	2013	USA	-	FED	29	88	-	-	6	6	4
Houman	2016	FR	-	Endothelial dysfunction	15	15/15*	-	-	6	6	4
Talajic	2014	USA	-	FED	34	30	-	-	6	6	4

DMEK = Descemet’s membrane endothelial keratoplasty; DSEK = Descemet’s stripping automated endothelial keratoplasty; ARVO = Association for Research and Vision in Ophthalmology; USA = United States of America; IN = India; GER = Germany; GR = Greece; CA = Canada; UK = United Kingdom; PRO = Portland; FR = France; R = retrospective design; P = prospective design; Fuchs = Fuchs’ dystrophy; PBK = pseudophakic bullous keratopathy; ICE = iridocorneal endothelial syndrome

*Routine DSEK/ultra-thin DSEK;

^#^ studies declaring collecting data during DMEK learning curve

### Efficacy analysis

#### Visual outcomes

**Fig 2:**
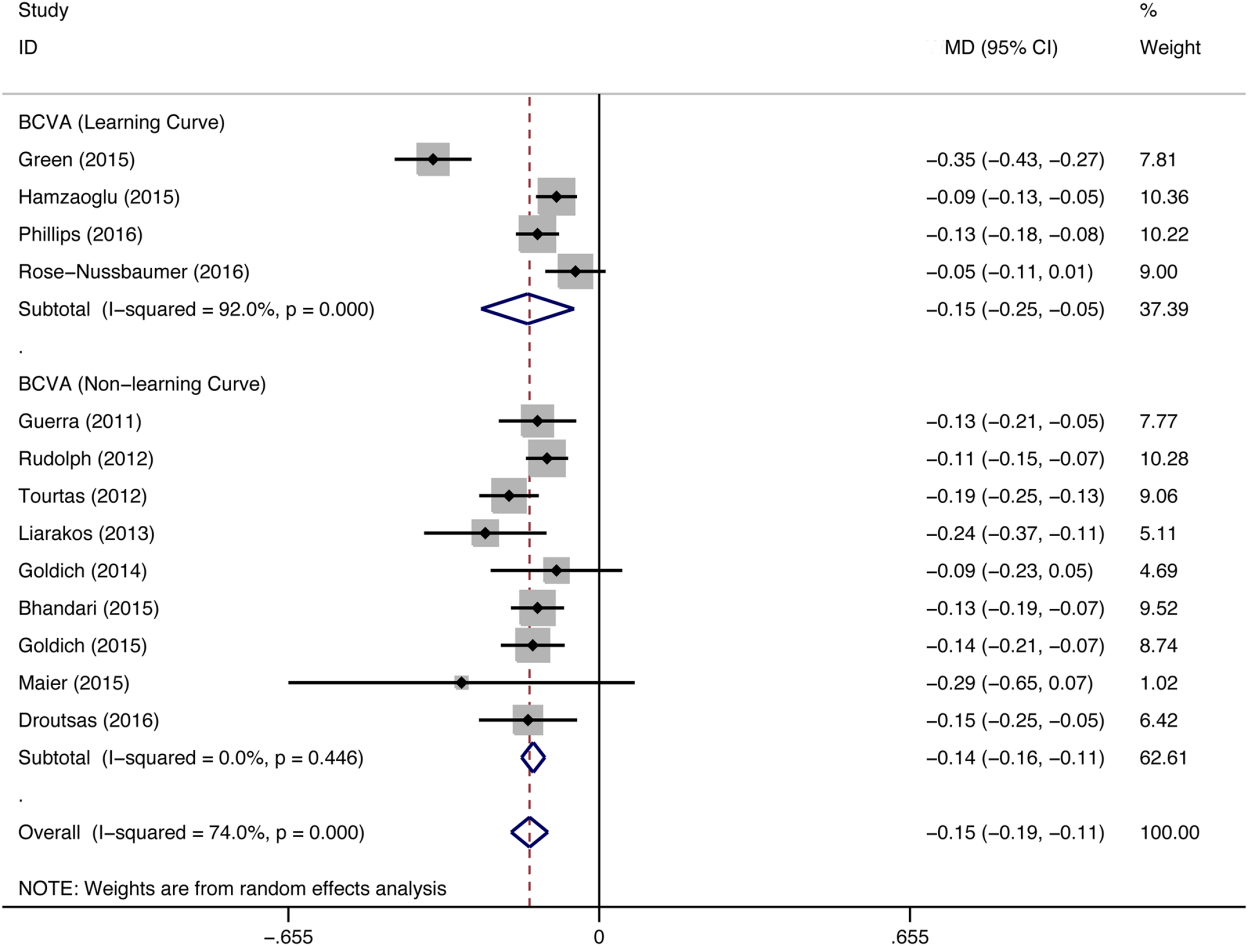
Forest plot comparing the postoperative best-corrected visual acuity (BCVA) between DMEK and DSEK. CI = confidence interval; SD = standard deviation; DMEK = Descemet’s membrane endothelial keratoplasty; DSEK = Descemet’s stripping endothelial keratoplasty. Random-effects model (standard DerSimonian-Laird) was used.

**Fig 3:**
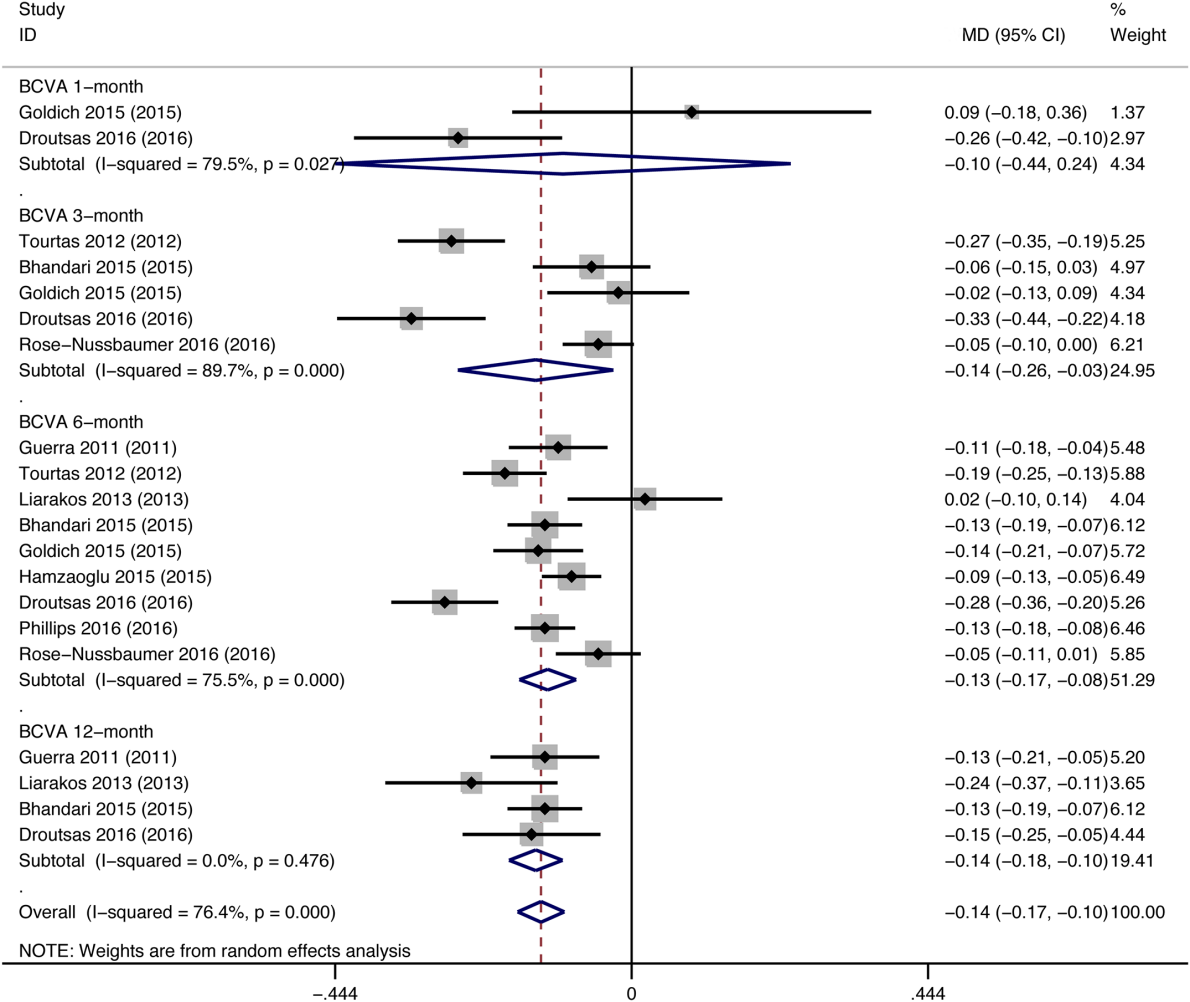
Forest plot comparing the postoperative best-corrected visual acuity (BCVA) between DMEK and DSEK at different follow-up period. CI = confidence interval; SD = standard deviation; DMEK = Descemet’s membrane endothelial keratoplasty; DSEK = Descemet’s stripping endothelial keratoplasty. Random-effects model (standard DerSimonian-Laird) was used.

Seventeen studies (DMEK/DSEK = 949/621 eyes) reported postoperative BCVA. Of these studies, 11 (DMEK/DSEK = 822/451 eyes) showed that DMEK yielded significantly better visual rehabilitation than DSEK, two (DMEK/DSEK = 59/35 eyes) showed better BCVA in DMEK eyes at one postoperative time point but not at the other, and the remaining 4 studies (DMEK/DSEK = 68/135 eyes) did not find a significant difference in the visual improvement between DMEK and DSEK. The final postoperative BCVA from 13 studies (DMEK/DSEK = 354/313 eyes) was used for the meta-analysis. The overall pooled data provided strong evidence for better BCVA after DMEK than DSEK (MD = -0.15, 95% CI = -0.19 to -0.11, P<0.001). Four studies (DMEK/DSEK = 172/158 eyes) reported data during the surgeons’ DMEK learning curve phase. However, the surgeons’ experience was not found to materially alter the comparison of visual outcomes between the two EK procedures (pooled data during DMEK learning curve: MD = -0.15, 95% CI = -0.25 to -0.05, P = 0.003; pooled data from the remaining studies: MD = -0.14, 95% CI = -0.16 to -0.11, P<0.001) (Fig 2). Moreover, BCVA was reported in different follow-up period. No significant difference between DMEK and DSEK was found at postoperative 1 month (MD = -0.10, 95% CI = -0.44 to 0.24, P = 0.555). However, DMEK yielded significant better visual outcomes compared with DSEK at postoperative 3-month, 6-month and 12-month (3-month: MD = -0.14, 95% CI = -0.26 to -0.03, p = 0.015; 6-month: MD = -0.13, 95% CI = -0.17 to -0.08, P<0.001; 12-month: MD = -0.14, 95% CI = -0.18 to -0.10, P<0.001) (Fig 3). The comparison outcomes were summarized in Table 2.

**Table 2:** Description of meta-analysis of outcomes between DMEK and DSEK.

Outcomes	No. of studies	No. of eyes		Effect estimates*	
		DMEK	DSEK	MD/OR/RD (95% CI)	P value
BCVA (learning curve)	4	172	158	-0.15 (-0.25, -0.05)	0.003
BCVA (non-learning curve)	9	182	155	-0.14 (-0.16, -0.11)	<0.001
BCVA (overall)	13	354	313	-0.15 (-0.19, -0.11)	<0.001
BCVA (1-month)	2	42	39	-0.10 (-0.44, 0.24)	0.555
BCVA (3-month)	5	150	116	-0.14 (-0.26, -0.03)	0.015
BCVA (6-month)	9	294	262	-0.13 (-0.17, -0.08)	<0.001
BCVA (12-month)	4	77	63	-0.14 (-0.18, -0.10)	<0.001
ECD (learning curve)	3	178	189	-361.24 (-649.42, -73.07)	0.014
ECD (non-learning curve)	7	139	119	177.61 (-2.40, 357.63)	0.053
ECD (overall)	10	317	308	14.88 (-181.50, 211.27)	0.882
ECD (3-month)	1	36	32	-280.00 (-445.92, -114.08)	<0.001
ECD (6-month)	10	320	309	25.59 (-183.15, 234.32)	0.810
ECD (12-month)	4	78	63	93.42 (-112.01, 298.85)	0.373
Graft detachment (learning curve)	5	266	250	3.42 (1.40, 8.36)	0.007
Graft detachment (non-learning curve)	5	94	84	4.66 (1.34, 16.21)	0.016
Graft detachment (overall)	10	360	334	4.56 (2.43, 8.58)	<0.001
Graft rejection	9	819	884	-0.04 (-0.08, -0.002)	0.042
Graft failure	5	271	245	0.03 (-0.01, 0.07)	0.171
High IOP	4	141	134	0.01 (-0.02, 0.04)	0.441
Tissue loss	2	45	45	0.04 (-0.04, 0.12)	0.352

BCVA = best corrected visual acuity; ECD = endothelial cell density; IOP = intraocular pressure; DMEK = Descemet’s membrane endothelial keratoplasty; DSEK = Descemet’s stripping automated endothelial keratoplasty; CI = confidence interval; MD = Mean difference; OR = odd ratio; RD = risk difference; N/A = not applicable.

*: Random-effects model (standard DerSimonian-Laird) was used.

### Safety analysis

#### Endothelial cell loss (ECL)

**Fig 4:**
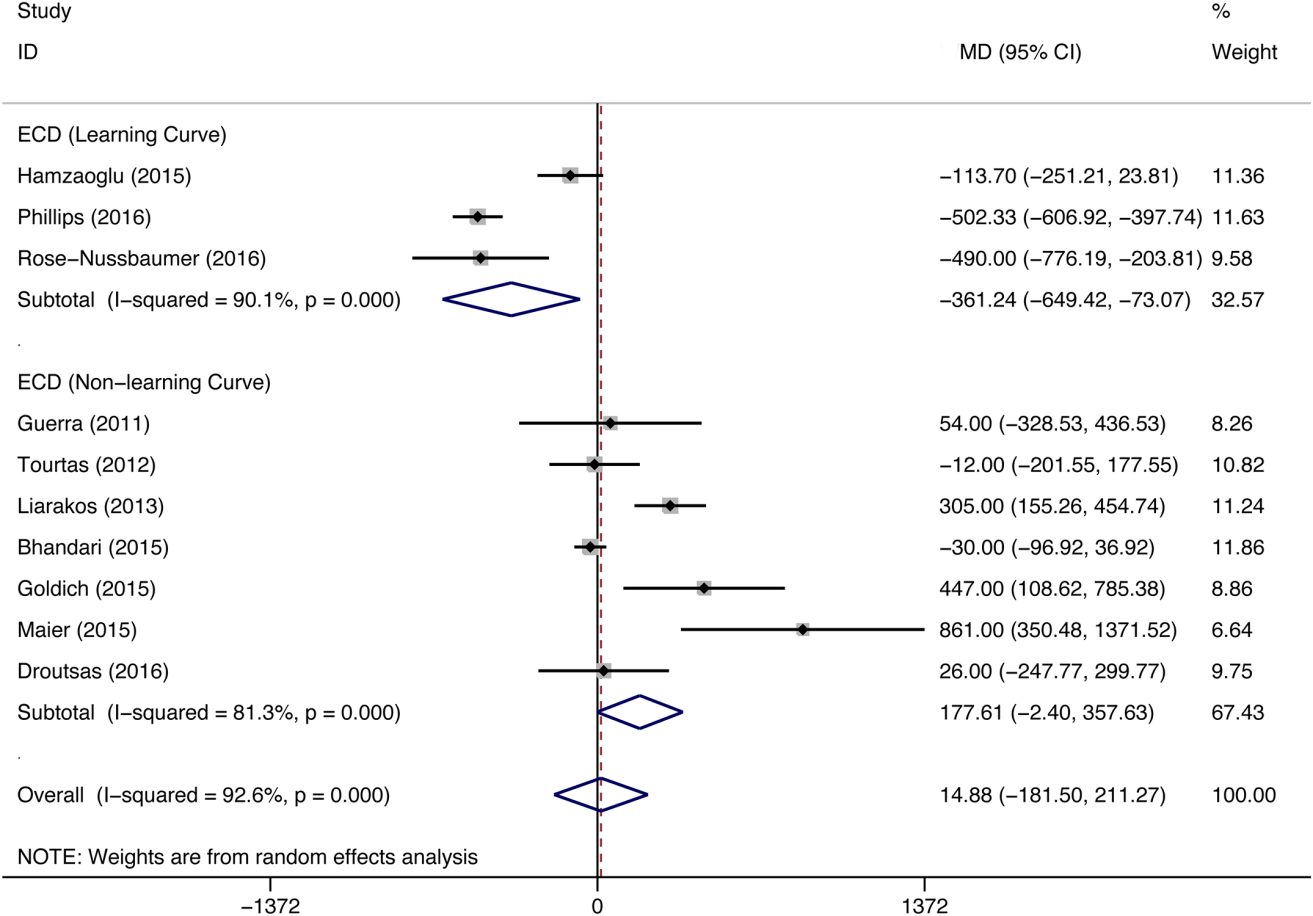
Forest plot comparing the postoperative endothelial cell density (ECD) between the DMEK and DSEK procedures. CI = confidence interval; SD = standard deviation; DMEK = Descemet’s membrane endothelial keratoplasty; DSEK = Descemet’s stripping endothelial keratoplasty. Random-effects model (standard DerSimonian-Laird) was used.

**Fig 5:**
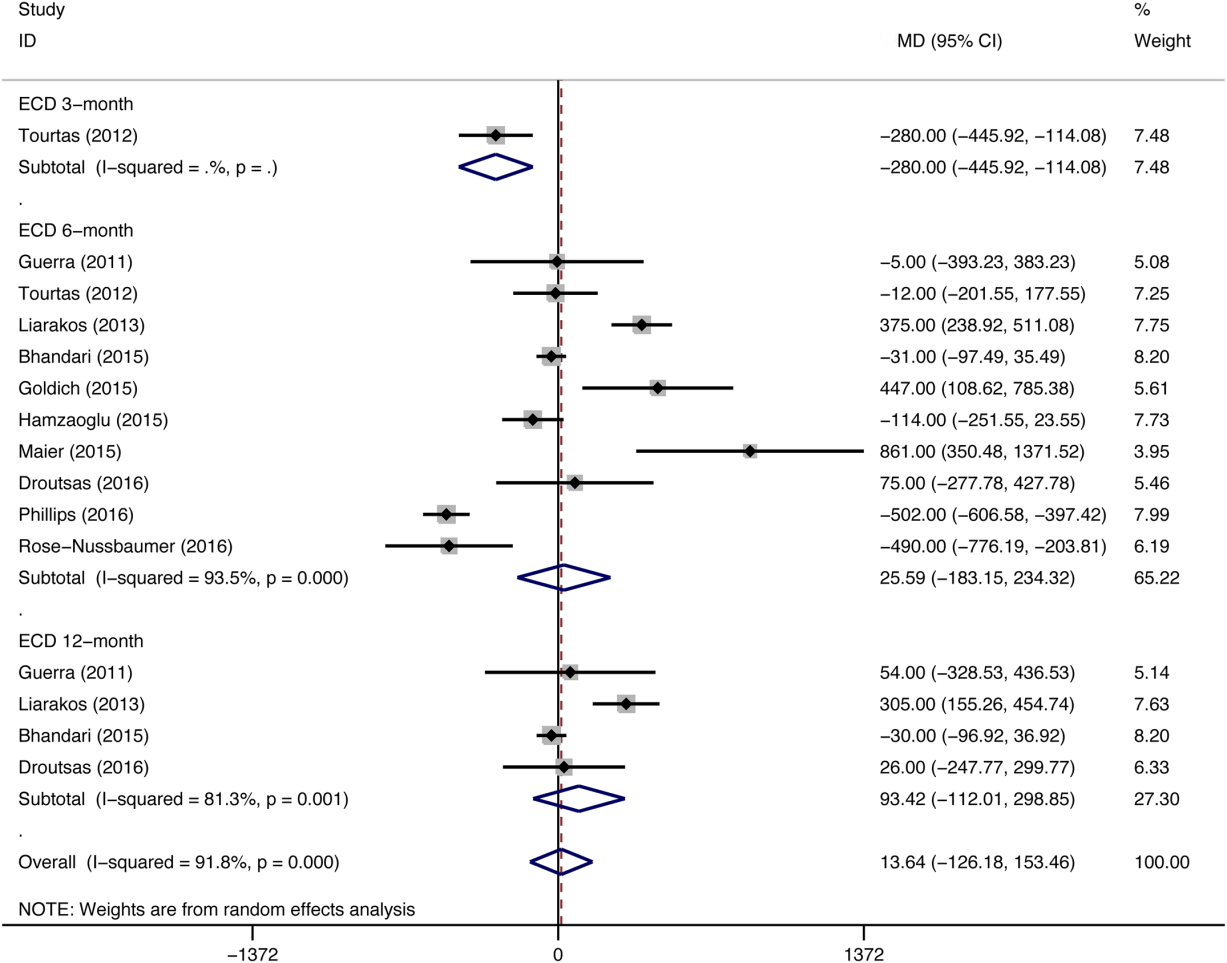
Forest plot comparing the postoperative endothelial cell density (ECD) between DMEK and DSEK at different follow-up period. CI = confidence interval; SD = standard deviation; DMEK = Descemet’s membrane endothelial keratoplasty; DSEK = Descemet’s stripping endothelial keratoplasty. Random-effects model (standard DerSimonian-Laird) was used.

A total of 11 eligible studies (DMEK/ DSEK = 767/397 eyes) compared the ECL between DMEK and DSEK. 8 studies (DMEK/DSEK = 608/262 eyes), including the study that enrolled patients with ocular comorbidities, did not observe differences in the postoperative ECD between the two surgeries; however, the remaining studies (DMEK/DSEK = 159/135 eyes) showed opposite results. The overall pooled estimates for the final postoperative ECD from 10 studies (DMEK/DSEK = 317/308 eyes) did not show significant differences between the DMEK and DSEK groups (MD = 14.88, 95% CI = -181.50 to 211.27, P = 0.882), and 3 studies (DMEK/DSEK = 178/189 eyes) presented data that had been collected during the surgeons’ DMEK learning curve phase. The pooled estimates from these 3 studies showed higher postoperative ECD in patients who had undergone DSEK than in patients who had undergone DMEK (MD = -361.24, 95% CI = -649.42 to -73.07, P = 0.014), whereas significant differences in the postoperative ECD were not observed between the DMEK and DSEK groups in the remaining studies (DMEK/DSEK = 139/119 eyes) (MD = 177.61, 95% CI = -2.40 to 357.63, P = 0.053) (Fig 4). ECD was reported in different follow-up period. No significant difference in postoperative ECD was found between DMEK and DSEK at 3-month, 6-month and 12-month after surgery (3-month: MD = -280.00, 95%CI = -445.92 to -114.08, P value was not estimable because only one study was included; 6-month: MD = 25.59, 95% CI = -183.15 to 234.32, P = 0.81; 12-month: MD = 93.42, 95% CI = -112.01 to 298.85, P = 0.373) (Fig 5).

#### Graft detachment

**Fig 6:**
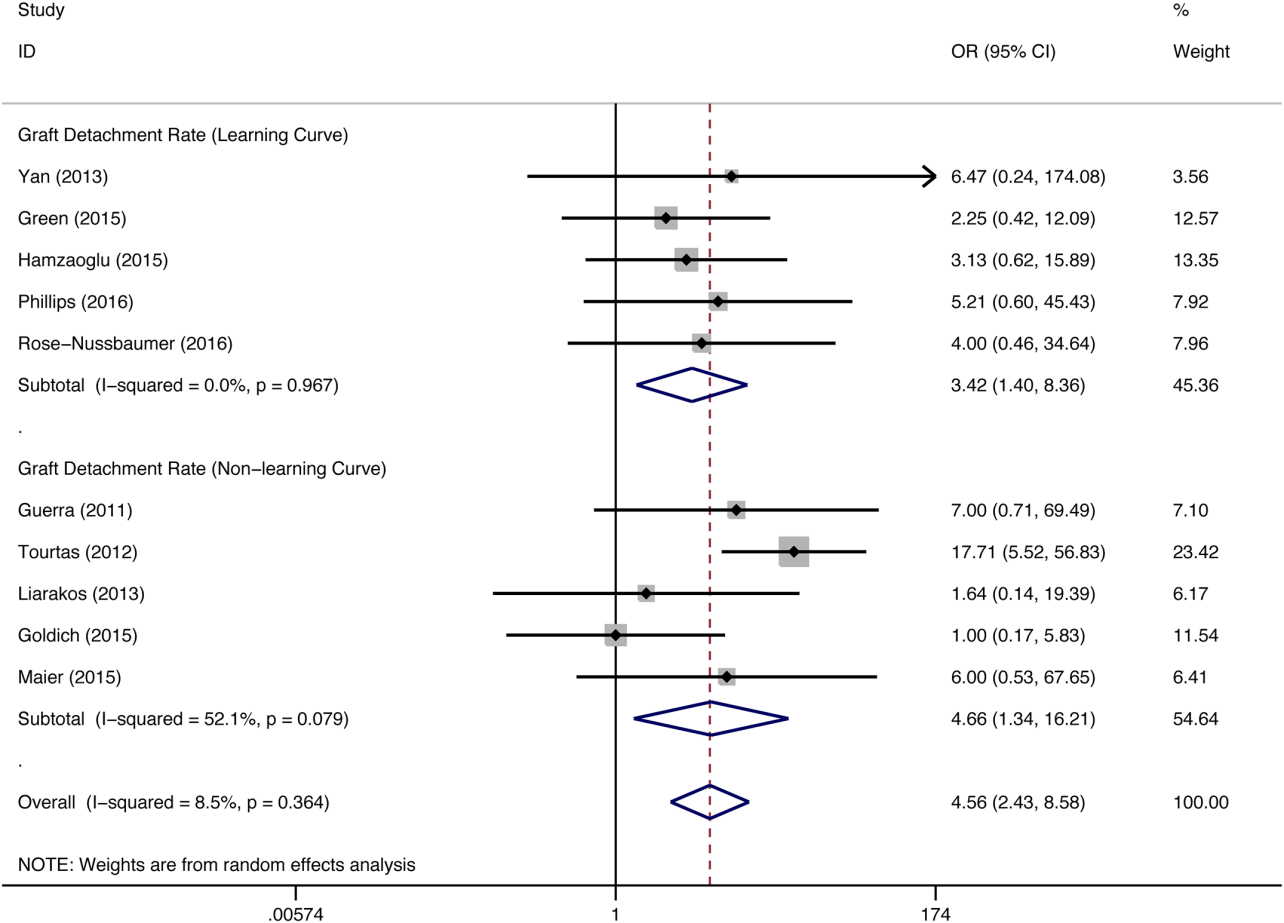
Forest plot comparing the graft detachment rate between DMEK and DSEK. CI = confidence interval; SD = standard deviation; DMEK = Descemet’s membrane endothelial keratoplasty; DSEK = Descemet’s stripping endothelial keratoplasty. Random-effects model (standard DerSimonian-Laird) was used.

For both the DMEK and DSEK groups, partial or total graft detachment that required rebubbling, repositioning or re-surgery was the most frequent complication. A total of 11 studies (DMEK/DSEK = 306/334 eyes) reported the graft detachment rate; this complication was manageable by rebubbling in most studies, repositioning was required in several studies, and re-surgery was seldom required (DMEK: 6% to 81.58%; DSEK: 2% to 27%). With the exception of one study (DMEK/DSEK = 17/17 eyes) that reported an equal graft detachment rate between DMEK and DSEK, the remaining 10 studies (DMEK/DSEK = 289/317 eyes) reported a higher graft detachment rate in the DMEK group than the DSEK group (overall pooled estimates: OR = 4.56, 95% CI = 2.43 to 8.58, P<0.001; pooled data during the DMEK learning curve: OR = 3.42, 95% CI = 1.40 to 8.36, P = 0.007; pooled data from the remaining studies: OR = 4.66, 95% CI = 1.34 to 16.21, P = 0.016) (Fig 6).

#### Graft rejection

**Fig 7:**
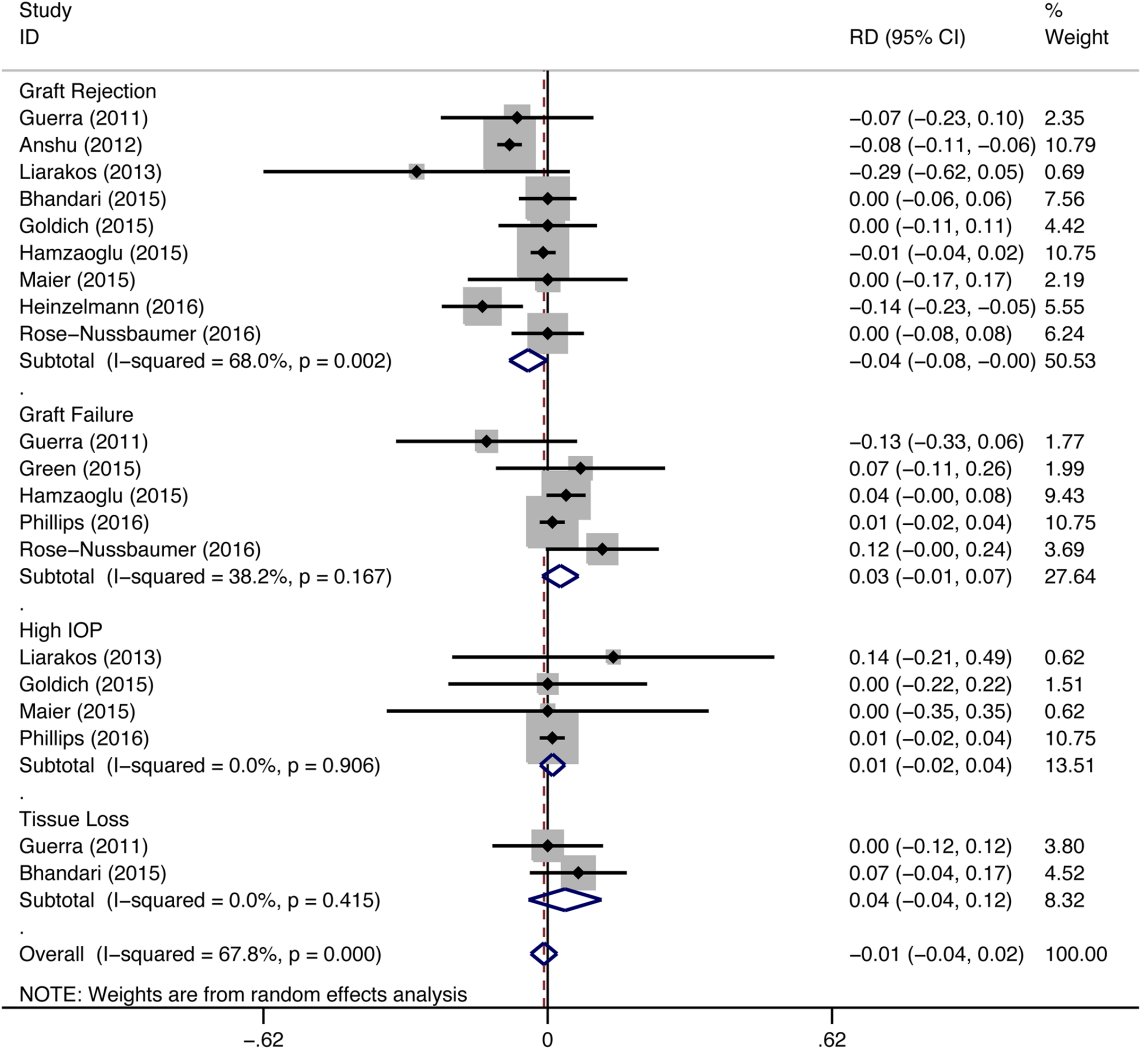
Forest plot comparing the surgical complications between DMEK and DSEK. CI = confidence interval; SD = standard deviation; DMEK = Descemet’s membrane endothelial keratoplasty; DSEK = Descemet’s stripping endothelial keratoplasty. Random-effects model (standard DerSimonian-Laird) was used.

Nine studies (DMEK/DSEK = 819/884 eyes) reported the incidence of graft rejection after EK. The DMEK group tended to have a slightly lower rejection rate than the DSEK group (risk difference (RD) = -0.04, 95% CI = -0.08 to -0.002; P = 0.042) (Fig 7).

#### Graft failure

Five studies (DMEK/DSEK = 217/245 eyes) reported the rate of graft failure during EK. The pooled data did not reveal significant differences in the incidence of graft failure between the DMEK and DSEK groups (RD = 0.03, 95% CI = –0.01 to 0.07, P = 0.171). (Fig 7)

#### Postoperative high intraocular pressure

High IOP during early postoperative period is good for graft tissue detachment. Postoperative high IOP could be caused by air bubbling, the topical use of steroids or exacerbating of preexisting glaucoma. Four studies (DMEK/DSEK = 141/134 eyes) reported high IOP after surgery. 7 out of 14 cases was due to air bubble-induced mechanical closure on the first postoperative day (5 cases in DMEK and 3 cases in DSEK), while steroid-induced high IOP occurred 1 DMEK eye and 2 DSEK eyes three months postoperatively. The pooled data did not show any significant differences in the overall incidence of postoperative IOP elevation between the two procedures (RD = 0.01, 95% CI = -0.02 to 0.04, P = 0.441). (Fig 7)

#### Tissue loss

Two studies (DMEK/DSEK = 45/45 eyes) reported the rate of tissue loss during graft preparation, and one of them did not observe any tissue loss during DMEK or DSEK graft preparation. Overall, the pooled estimates for the rate of tissue loss did not reveal significant differences between DMEK and DSEK (RD = 0.04, 95% CI = -0.04 to 0.12, P = 0.352). (Fig 7)

### Heterogeneity and publication bias

A great between-study heterogeneity was found based on the Q statistic, and *I*^*2*^ or *H*^*2*^ statistic (Table 3 and S2 Table). Nevertheless, the sensitivity analysis showed that the exclusion of any single study did not significantly change the overall pooled estimates, except one study [35]. Removal of this study leaded to a significant change in the overall pooled estimates for postoperative ECD. However, of note, with including this study or not, the pooled effects estimates consistently showed better visual recovery, comparable ECL and higher graft detachment rate in DMEK compared to DSEK.

**Table 3:** Description of between-study heterogeneity*.

Outcomes	No. Study	*I*^*2*^ *(95% CIs)*	*H*^*2*^ (95% CIs)	P-Value
BCVA (learning curve)	4	92% (83%, 96%)	12.55 (5.81, 27.10)	<0.001
BCVA (non-learning curve)	9	0% (0%, 65%)	1.00 (0.35, 2.84)	0.446
BCVA (overall)	13	74% (55%, 85%)	3.84 (2.22, 6.66)	<0.001
BCVA (1-month)	2	79% (11%, 95%)	4.87 (1.13, 21.04)	0.027
BCVA (3-month)	5	90% (79%, 95%)	9.74 (4.73, 20.04)	<0.001
BCVA (6-month)	9	76% (53%, 87%)	4.09 (2.12, 7.87)	<0.001
BCVA (12-month)	4	0% (0%, 85%)	1.00 (0.15, 6.53)	0.476
ECD (learning curve)	3	90% (74%, 96%)	10.08 (3.78, 26.89)	<0.001
ECD (non-learning curve)	7	81% (62%, 91%)	5.33 (2.65, 10.75)	<0.001
ECD (overall)	10	93% (89%, 95%)	13.56 (8.69, 21.14)	<0.001
ECD (3-month)	1	N/A	N/A	N/A
ECD (6-month)	10	94% (90%, 96%)	15.49 (10.11, 23.74)	<0.001
ECD (12-month)	4	81% (51%, 93%)	5.35 (2.05, 13.95)	0.001
Graft detachment (learning curve)	5	0% (0%, 79%)	1.00 (0.21, 4.81)	0.967
Graft detachment (non-learning curve)	5	52% (0%, 82%)	2.09 (0.77, 5.68)	0.079
Graft detachment (overall)	10	8% (0%, 65%)	1.09 (0.41, 2.90)	0.364
Graft rejection	9	68% (36%, 84%)	3.13 (1.55, 6.28)	<0.001
Graft failure	5	38% (0%, 77%)	1.62 (0.60, 4.35)	0.15
High IOP	4	0% (0%, 85%)	1.00 (0.15, 6.53)	0.852
Tissue loss	2	0% (0%, 100%)	1.00 (N/A)	0.405

BCVA = best corrected visual acuity; ECD = endothelial cell density; IOP = intraocular pressure; CI = confidence interval; N/A = not applicable.

*: Random-effects model (standard DerSimonian-Laird) was used.

**Fig 8:**
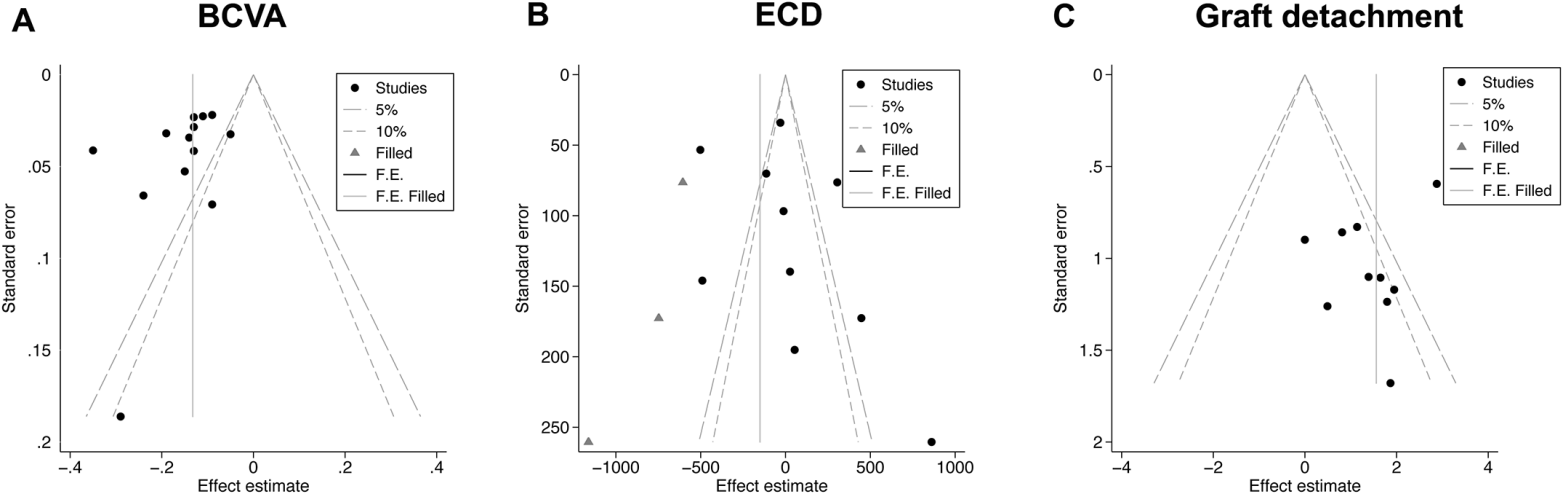
Contour enhanced funnel plots assessing the potential impact of publication bias. (A, B) No study need be filled or trimmed. (C) 3 studies were filled, but these 3 studies were located in the regions of statistical significance on funnel, indicating that plot asymmetry was not caused by publication bias. BCVA = best corrected visual acuity; ECD = endothelial cell density; MD = mean difference; OR = odds ratio; se = standard error.

Publication bias were only performed for primary outcomes, including the postoperative BCVA, ECD and graft detachment rate, because the secondary outcomes analysis included less than 10 studies. Neither contour-enhanced funnel plots, nor the Begg rank correlation test, the Egger or Harbord liner regression test revealed any publication bias (postoperative BCVA, ECD and graft detachment rate: Begg’s Test: P = 0.077, 0.474, 0.721; Egger’s Test: P = 0.179, 0.444, 0.254; respectively. Horbard Test: P = 0.208 for graft detachment rate. Fig 8).

Publication years were also considered in bias assessment. Cumulative meta-analysis was performed by sequentially adding studies into the analysis based on the publication years. In the cumulative forest plots, we found a consistent, statistically significant better visual recovery, but higher graft detachment rate and equivalent endothelia cell loss in DMEK compared to DSAK (S1–S3 Figs). Neither was “Proteus” phenomenon over years found among studies (S4–S6 Figs).

## Discussion

In this comprehensive, systematic review of all available and eligible studies, the results showed that DMEK provides better visual rehabilitation than DSEK, even when DMEK was performed by a surgeon during the learning curve phase. Nevertheless, less surgical experience with DMEK did materially influence the ECL between the two EK procedures, which resulted in more ECL in the DMEK group than in the DSEK group. Although the findings indicated that DMEK is a safe alternative for DSEK, a higher rebubbling rate represented a major concern for DMEK.

In the DSEK procedure, damaged cells are replaced with a donor button that consists of endothelium, Descemet’s membrane and posterior stroma[15]. As a sutureless keratoplasty, DSEK is a more precise treatment and is associated with better overall surgical outcomes than conventional penetrating keratoplasty (PK) conditions such as Fuchs’ dystrophy[41–43]. However, DSEK is an additive procedure, and the corneas become thicker in DSEK eyes, which contributes to the postoperative hyperopic refractive shift[44, 45]. The extra stroma in DSEK grafts and the rough recipient-donor interface increase the posterior irregular astigmatism and uncorrectable corneal high-order aberrations (HOAs), which limit the visual outcomes[22]. In contrast, DMEK uses an extremely thin graft that consists of only Descemet’s membrane and endothelium without adherent donor stroma[1]. This promising new procedure leads to better restorations of the corneal integrity, eliminates recipient-donor interface mismatches, and allows for better visual outcomes; thus, it has become popular among corneal surgeons and patients who suffer from corneal endothelial diseases[11–13, 16, 18–22].

Many studies have compared the visual outcomes between DMEK and DSEK and reported that DMEK results in better visual acuity than DSEK[11–13, 20, 22, 31, 33, 34], which is similar to the results of our meta-analysis. Nevertheless, several studies did not find a significant difference in terms of the postoperative BCVA between the two EK surgeries[18, 30, 36]. These variations might have been caused by differences in the graft thickness used in the DSEK procedure and differences in the surgeons’ technical skills. Graft thickness might affect functional outcomes in DSEK[44]. Although a recent meta-analysis indicated that graft thickness was not correlated with BCVA after DSEK, a well-designed multicenter RCT and many other studies showed that ultra-thin DSEK (UT-DSAEK) provides better visual outcomes than routine DSEK[39, 46–48]. Moreover, despite the favorable visual outcomes achieved by DMEK, the long learning curve still prevents many surgeons from embracing DMEK[5]. Therefore, we performed a meta-analysis on studies that collected data during the DMEK learning curve and a separate analysis on the remaining studies. The pooled estimates from both subgroups supported better visual rehabilitation after DMEK than DSEK. This finding might encourage surgeons to convert to DMEK.

DSEK uses a “precut” graft that is “pre-stripped” by tools such as microtomes and femtosecond lasers; however, DMEK presents an inexpensive, but more challenging graft preparation procedure. DMEK grafts are extremely thin and fragile and may be stretched, folded or ruptured during preparation. In addition, unfolding and placing a DMEK graft in the anterior chamber is more difficult than manipulating a DSEK graft. Although DMEK graft preparation and manipulation presented difficulties, our pooled data showed equivalent ECL between DMEK and DSEK. However, the separate meta-analyses performed on studies that collected data during the DMEK learning curve phase indicated a higher rate of ECL after DMEK than after DSEK. This finding may indicate that the ECL associated with graft preparation and manipulation was reduced with experience after the surgeons overcame the technique learning curve.

For both EK procedures, graft detachment in the early postoperative course is among the most commonly reported problems. Our pooled data showed a significantly higher incidence of graft detachment that required rebubbling or repositioning in DMEK than in DSEK, even when the data collected during the surgeons’ learning curve were excluded. The graft detachment rates in the DMEK and DSEK procedures varied substantially among the studies [11–13, 16, 19–21, 49]. A graft can be positioned in place with air or with a long-lasting agent, such as 20% sulfur hexafluoride (SF6). Güell et al. found that tamponade with 20% SF6 yielded a significantly lower incidence of graft detachment in DMEK than with the use of 100% air[50]. Among the eligible studies included in our meta-analysis, 3 studies[20, 21, 35] reported using 20% SF6 for DMEK graft detachment and 6 studies[11, 13, 16, 19, 30, 36] used 100% air in the DMEK procedure. Correspondingly, the DMEK graft detachment for the 20% SF6 and 100% air tamponade ranged from 5% to 42.68% and from 17.65% to 81.58%, respectively. Moreover, the amount of gas injected, the duration of a complete gas fill, the patient’s position and the surgeon’s skills might have contributed to the variations in graft detachment.

Most graft detachment is manageable via one or repeated intracameral rebubbling procedures. When and whether to rebubble is generally arbitrarily decided by the surgeons. Because most partially dislocated DSAEK grafts will be spontaneously reattached, in most cases, rebubbling is only performed for full dislocation[51]. Although the complete detachment of a DMEK graft is rare, thin and fragile DMEK grafts are actually extremely difficult to reattach[51]. Therefore, surgeons might be more willing to perform an intracameral air injection for partially dislocated DMEK grafts than for DSEK grafts.

The incidence of other complications, including immune rejection, postoperative high IOP, graft failure, tissue loss, etc., were low for both the DMEK and DSEK groups. Immune rejection remains the leading cause of long-term graft failure after keratoplasty despite the relative immune privilege of the cornea. Compared with DSEK, DMEK uses a graft without antigenic donor stroma[52]. Therefore, the risk of graft rejection should be minimized in DMEK. Consistently, in our meta-analysis, our pooled data also show a significant, but only slightly lower rejection rate for DMEK than in DSEK. However, as for graft rejection, there is substantial discordance among studies, which was likely because of the low rejection rate in both EK procedures or the various follow-up duration for the included studies.

Our study had several potential limitations. First, most of the included studies in our meta-analysis were small retrospective studies rather than prospective clinical studies, which might have negatively affected the veracity of our meta-analysis because of possible selection bias, reporting bias and other confounding bias. Future well-designed RCTs with large sample sizes and an extensive follow-up duration are needed to reach definitive conclusions. Second, our meta-analysis only included limited number of studies. In this case, all statistic methods used in our study, such as publication bias tests and heterogeneity assessment, might be underpowered to detect the significance, and thus possibly tend to result in unjustified conclusions. Thirdly, substantial between-study heterogeneity was observed in our study. To reduce the heterogeneity, subgroup analysis was performed according to surgeons’ skills and follow-up time. Some of the heterogeneity was found to be substantially reduced following subgroup analysis (e.g. the *I*^*2*^ statistic of BCVA in non-learning curve and postoperative 12-month subgroups were reduced to 0%), but high between-study heterogeneity still existed. We would likely to attribute these heterogeneities to differences in the operative technical details, surgeon skill, or follow-up durations.

## Conclusions

In conclusion, the current data indicate that despite the higher incidence of graft detachment, the DMEK procedure yielded better visual acuity than the DSEK procedure and equivalent ECL outcomes and overall operative complications. The learning curve does not appear to materially affect the superiority of DMEK over DSEK in terms of the visual outcomes, which might encourage more surgeons to embrace DMEK.

## Supporting information

S1 PRISMA checklist (DOC)

S1 Table Random effects meta-analysis by traditional and more robust approaches. (DOCX)

S2 Table Description of between-study heterogeneity assessment using different random-effects approaches. (DOCX)

S1 Fig Cumulative meta-analysis of postoperative best corrected visual acuity (BCVA) based on publication years. ES = effects estimates; CI = confidence interval.
(TIF)

S2 Fig Cumulative meta-analysis of postoperative endothelial cell density (ECD) based on publication years. ES = effects estimates; CI = confidence interval.
(TIF)

S3 Fig Cumulative meta-analysis of graft detachment rate based on publication years. ES = effects estimates; CI = confidence interval.
(TIF)

S4 Fig Metatrend analysis of postoperative best corrected visual acuity (BCVA) based on publication years. First vs Subsequent method: P = 0.690. GLS regression-based test: including all studies: Coef. = -0.00042, P = 0.612; excluding first studies: Coef. = 0.00107, P = 0.398. ES = effects estimates.
(TIF)

S5 Fig Metatrend analysis of postoperative endothelial cell density (ECD) based on publication years. First vs Subsequent method: P = 0.850. GLS regression-based test: including all studies: Coef. = 1.249, P = 0.903; excluding first studies: Coef. = 1.136, P = 0.924. ES = effects estimates.
(TIF)

S6 Fig Metatrend analysis of graft detachment rate based on publication years. First vs Subsequent method: P = 0.686. GLS regression-based test: including all studies: Coef. = -0.137, P<0.001; excluding first studies: Coef. = -0.168, P<0.001. ES = effects estimates.
(TIF)
