# Supplementary material for: Efficacy and safety of Descemet’s membrane endothelial keratoplasty versus Descemet’s stripping endothelial keratoplasty: A systematic review and meta-analysis
Source: PLoS One. 2017 Dec 18;12(12):e0182275. doi: 10.1371/journal.pone.0182275 (PMC5734733; doi:10.1371/journal.pone.0182275)
Supplement: S1 Table — (DOCX) [file pone.0182275.s002.docx]

**S1 Table Random effects meta-analysis by traditional and more robust approaches**

| **Outcomes** | **Standard D-L** | | **Bootstrap D-L** | | | **Profile Likelihood** | | |
| --- | --- | --- | --- | --- | --- | --- | --- | --- |
|  | **Effects** | **95% CIs** | **Effects** | **95% CIs** | **Effects** | | **95% CIs** |  |
| BCVA (learning curve) | -0.15 | -0.25 to -0.05 | -0.15 | -0.26 to -0.05 | -0.15 | | -0.30 to -0.01 |  |
| BCVA (non-learning curve) | -0.14 | -0.16 to -0.11 | -0.14 | -0.17 to -0.11 | -0.14 | | -0.17 to -0.11 |  |
| BCVA (overall) | -0.15 | -0.19 to -0.11 | -0.15 | -0.19 to -0.11 | -0.15 | | -0.19 to -0.10 |  |
| BCVA (1-month) | -0.10 | -0.44 to 0.24 | -0.10 | -0.47 to 0.27 | -0.12 | | -0.51 to 0.33 |  |
| BCVA (3-month) | -0.14 | -0.26 to -0.03 | -0.14 | -0.26 to -0.03 | -0.14 | | -0.28 to -0.01 |  |
| BCVA (6-month) | -0.13 | -0.17 to -0.08 | -0.13 | -0.17 to -0.08 | -0.13 | | -0.18 to -0.07 |  |
| BCVA (12-month) | -0.14 | -0.18 to -0.10 | -0.15 | -0.19 to -0.10 | -0.14 | | -0.20 to -0.10 |  |
| ECD (learning curve) | -361.24 | -649.41 to -73.07 | -361.18 | -647.91 to -74.45 | -357.30 | | N/A |  |
| ECD (non-learning curve) | 177.61 | -2.40 to 357.63 | 182.30 | -8.94 to 373.53 | 173.36 | | N/A |  |
| ECD (overall) | 14.88 | -181.50 to 211.27 | 17.36 | -187.49 to 222.21 | 20.61 | | N/A |  |
| ECD (3-month) | -280.00 | -445.92 to -114.08 | -280.00 | -445.92 to -114.08 | N/A | | N/A |  |
| ECD (6-month) | 25.59 | -183.15 to 234.32 | 27.71 | -189.51 to 244.94 | 29.62 | | N/A |  |
| ECD (12-month) | 93.42 | -112.01 to 298.85 | 93.40 | -113.84 to 300.65 | 92.54 | | N/A |  |
| Graft detachment (learning curve) | 3.42 | 1.40 to 8.36 | 3.42 | 1.40 t0 8.36 | 3.42 | | 1.40 to 8.42 |  |
| Graft detachment (non-learning curve) | 4.66 | 1.34 to 16.21 | 4.70 | 1.40 to 15.83 | 4.85 | | 1.17 to 16.77 |  |
| Graft detachment (overall) | 4.56 | 2.43 to 8.58 | 4.41 | 2.24 to 8.68 | 4.37 | | 1.99 to 8.88 |  |
| Graft rejection | -0.04 | -0.08 to -0.002 | -0.04 | -0.08 to -0.001 | -0.04 | | -0.09 to -0.001 |  |
| Graft failure | 0.03 | -0.01 to 0.07 | 0.03 | -0.02 to 0.08 | 0.02 | | -0.00 to 0.06 |  |
| High IOP | 0.01 | -0.01 to 0.04 | 0.01 | -0.01 to 0.04 | 0.01 | | -0.03 to 0.06 |  |
| Tissue loss | 0.04 | -0.04 to 0.12 | 0.04 | -0.04 to 0.12 | 0.04 | | -0.07 to 0.14 |  |

BCVA = best corrected visual acuity; ECD = endothelial cell density; IOP = intraocular pressure; CI = confidence interval; N/A = not applicable; D-L= DerSimonian-Laird
