# Supplementary material for: Efficacy and safety of Descemet’s membrane endothelial keratoplasty versus Descemet’s stripping endothelial keratoplasty: A systematic review and meta-analysis
Source: PLoS One. 2017 Dec 18;12(12):e0182275. doi: 10.1371/journal.pone.0182275 (PMC5734733; doi:10.1371/journal.pone.0182275)
Supplement: S2 Table — (DOCX) [file pone.0182275.s003.docx]

**S2 Table Description of meta-analysis of outcomes using different random-effects approaches**

BCVA = best corrected visual acuity; ECD = endothelial cell density; IOP = intraocular pressure; CI = confidence interval; N/A = not applicable;

| **Outcomes** | **Bootstrap D-L** | | | **Profile Likelihood** | | |
| --- | --- | --- | --- | --- | --- | --- |
|  | *I^2^* (95% CIs) | *H^2^* (95% CIs) | P Value | *I^2^* (95% CIs) | *H^2^* (95% CIs) | P Value |
| BCVA (learning curve) | 93% (85%, 97%) | 14.18 (6.74, 29.84) | <0.001 | 94% (87%, 97%) | 15.62 (7.57, 32.20) | <0.001 |
| BCVA (non-learning curve) | 12% (0%, 54%) | 1.14 (0.60, 2.15) | 0.439 | 1% (0%, 65%) | 1.01 (0.35, 2.86) | 0.446 |
| BCVA (overall) | 75% (56%, 85%) | 3.95 (2.29, 6.81) | <0.001 | 77% (61%, 87%) | 4.35 (2.56, 7.41) | <0.001 |
| BCVA (1-month) | 82% (23%, 96%) | 5.52 (1.31, 23.34) | 0.026 | 57% (0%, 90%) | 2.32 (0.56, 9.71) | 0.027 |
| BCVA (3-month) | 90% (79%, 95%) | 9.87 (4.81, 20.26) | <0.001 | 86% (75%, 94%) | 8.60 (4.08, 18.15) | <0.001 |
| BCVA (6-month) | 77% (53%, 88%) | 4.28 (2.24, 8.17) | <0.001 | 78% (58%, 88%) | 4.49 (2.37, 8.51) | <0.001 |
| BCVA (12-month) | 20% (0%, 88%) | 1.24 (0.19, 8.13) | 0.473 | 0% (0%, 85%) | 1.00 (0.15, 6.53) | 0.476 |
| ECD (learning curve) | 88% (65%, 96%) | 8.09 (2.87, 22.80) | <0.001 | 82% (45%, 94%) | 5.60 (1.82, 17.25) | <0.001 |
| ECD (non-learning curve) | 81% (61%, 90%) | 5.18 (2.56, 10.49) | <0.001 | 79% (57%, 90%) | 4.76 (2.31, 9.78) | <0.001 |
| ECD (overall) | 92% (88%, 95%) | 12.81 (8.15, 20.13) | <0.001 | 94% (91%, 96%) | 16.86 (11.12, 25.57) | <0.001 |
| ECD (3-month) | N/A | N/A | N/A | N/A | N/A | N/A |
| ECD (6-month) | 93% (86%, 96%) | 14.53 (9.40, 22.45) | <0.001 | 95% (92%, 96%) | 18.32 (12.20, 27.49) | <0.001 |
| ECD (12-month) | 77% (36%, 91%) | 4.29 (1.57, 11.73) | 0.001 | 69% (9%, 89%) | 3.18 (1.10, 9.21) | 0.001 |
| Graft detachment (learning curve) | 0% (0%, 79%) | 1.00 (0.21, 4.81) | 0.968 | 0% (0%, 79%) | 1.00 (0.21, 4.81) | 0.967 |
| Graft detachment (non-learning curve) | 48% (0%, 81%) | 1.94 (0.71, 5.29) | 0.084 | 41% (0%, 78%) | 1.71 (0.63, 4.64) | 0.079 |
| Graft detachment (overall) | 18% (0%, 59%) | 1.22 (0.61, 2.43) | 0.359 | 22% (0%, 62%) | 1.28 (0.63, 2.61) | 0.364 |
| Graft rejection | 62% (21%, 82%) | 2.63 (1.27, 5.43) | 0.001 | 58% (13%, 80%) | 2.41 (1.15, 5.04) | 0.002 |
| Graft failure | 46% (0%, 80%) | 1.85 (0.68, 5.04) | 0.168 | 0% (0%, 79%) | 1.00 (0.21, 4.81) | 0.167 |
| High IOP | 0% (0%, 85%) | 1.00 (0.15, 6.53) | 0.907 | 0% (0%, 85%) | 1.00 (0.15, 6.53) | 0.906 |
| Tissue loss | 0% (0%, 100%) | 1.00 (N/A) | 0.414 | 0% (0%, 100%) | 1.00 (N/A) | 0.415 |

BCVA = best corrected visual acuity; ECD = endothelial cell density; IOP = intraocular pressure; CI = confidence interval; N/A = not applicable; D-L= DerSimonian-Laird
