# Supplementary figures and images for: Efficacy and safety of Descemet’s membrane endothelial keratoplasty versus Descemet’s stripping endothelial keratoplasty: A systematic review and meta-analysis
Source: PLoS One. 2017 Dec 18;12(12):e0182275. doi: 10.1371/journal.pone.0182275 (PMC5734733; doi:10.1371/journal.pone.0182275)

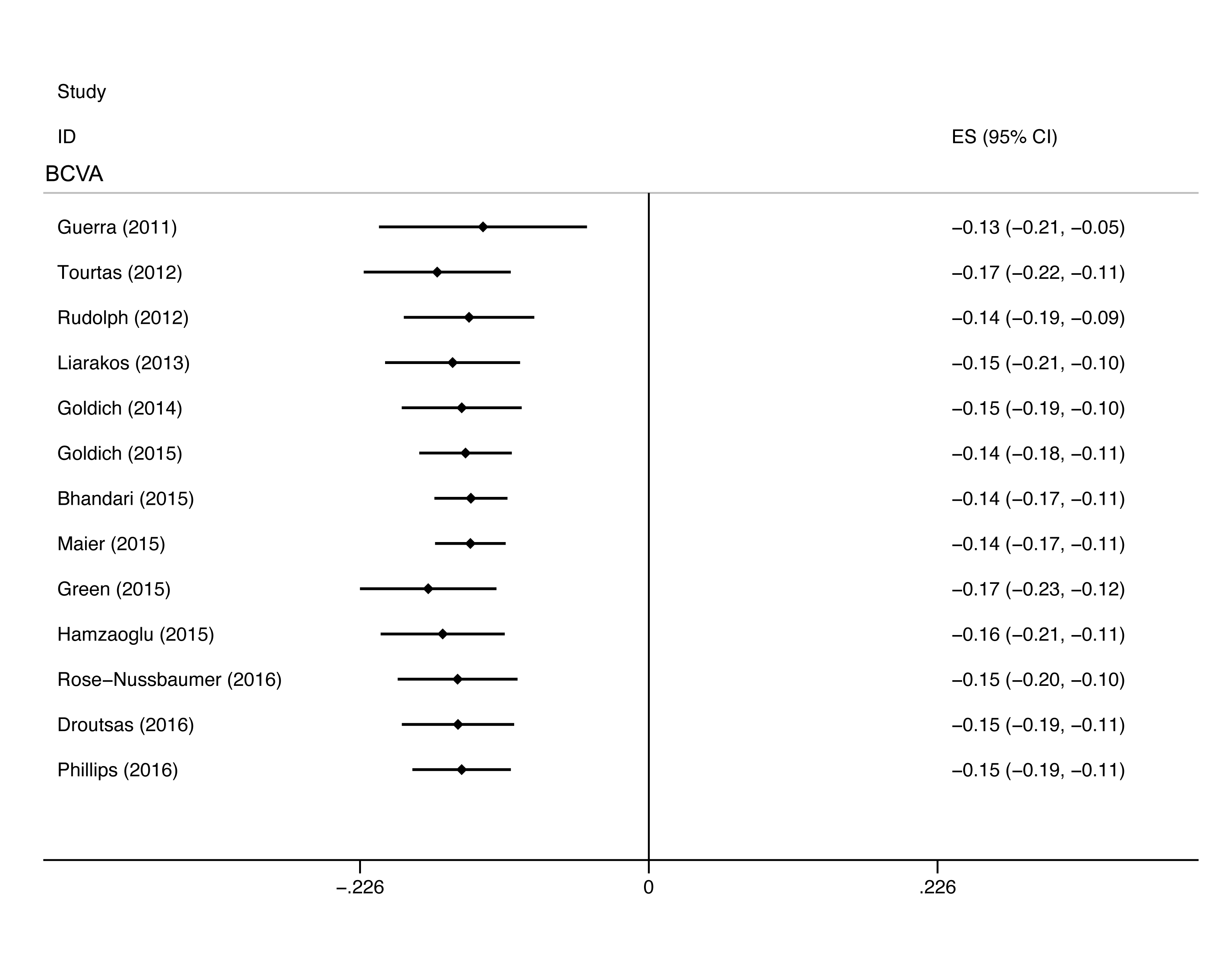

Supplement: S1 Fig — ES = effects estimates; CI = confidence interval. (TIF) [file pone.0182275.s004.tif]

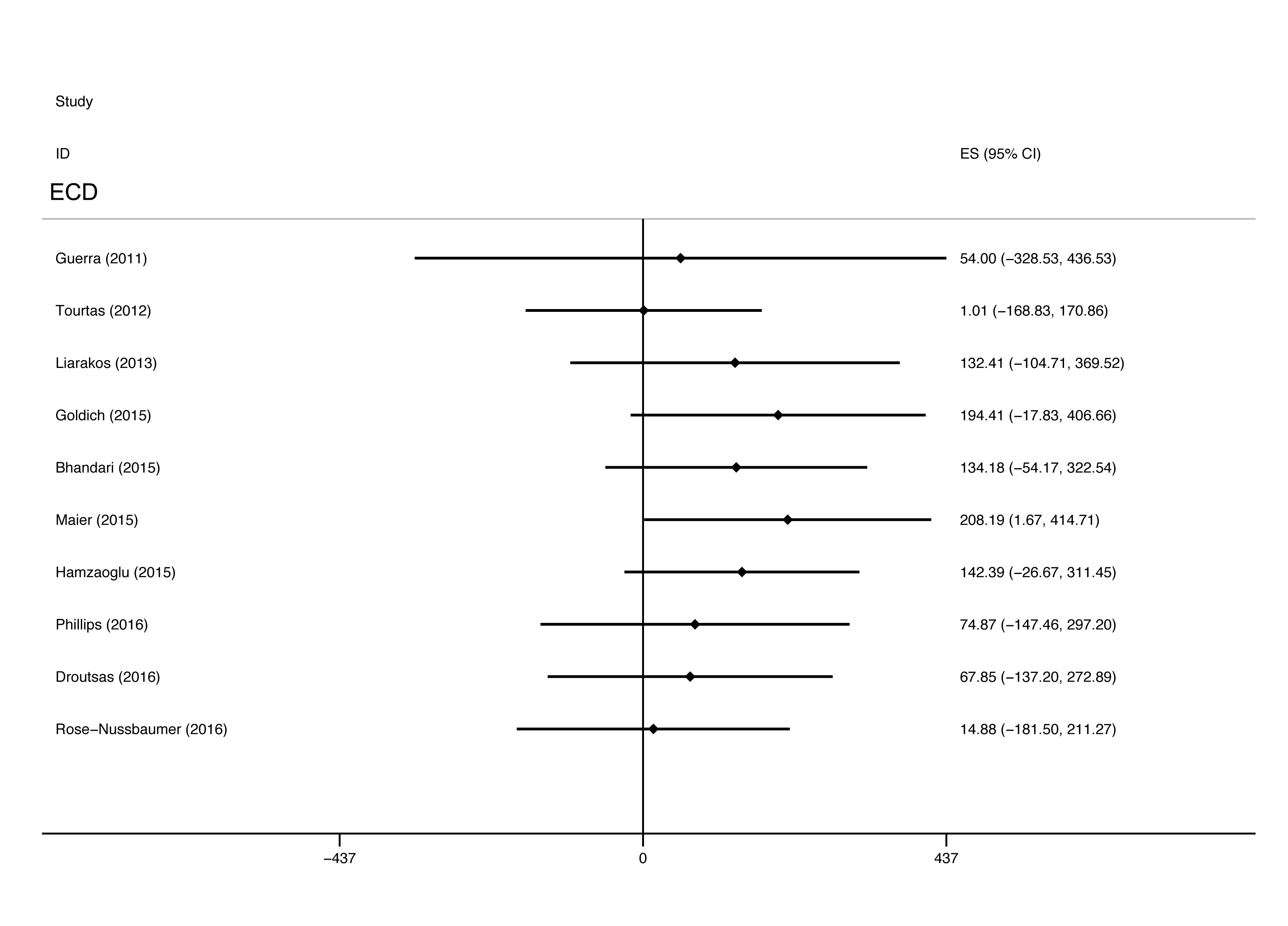

Supplement: S2 Fig — ES = effects estimates; CI = confidence interval. (TIF) [file pone.0182275.s005.tif]

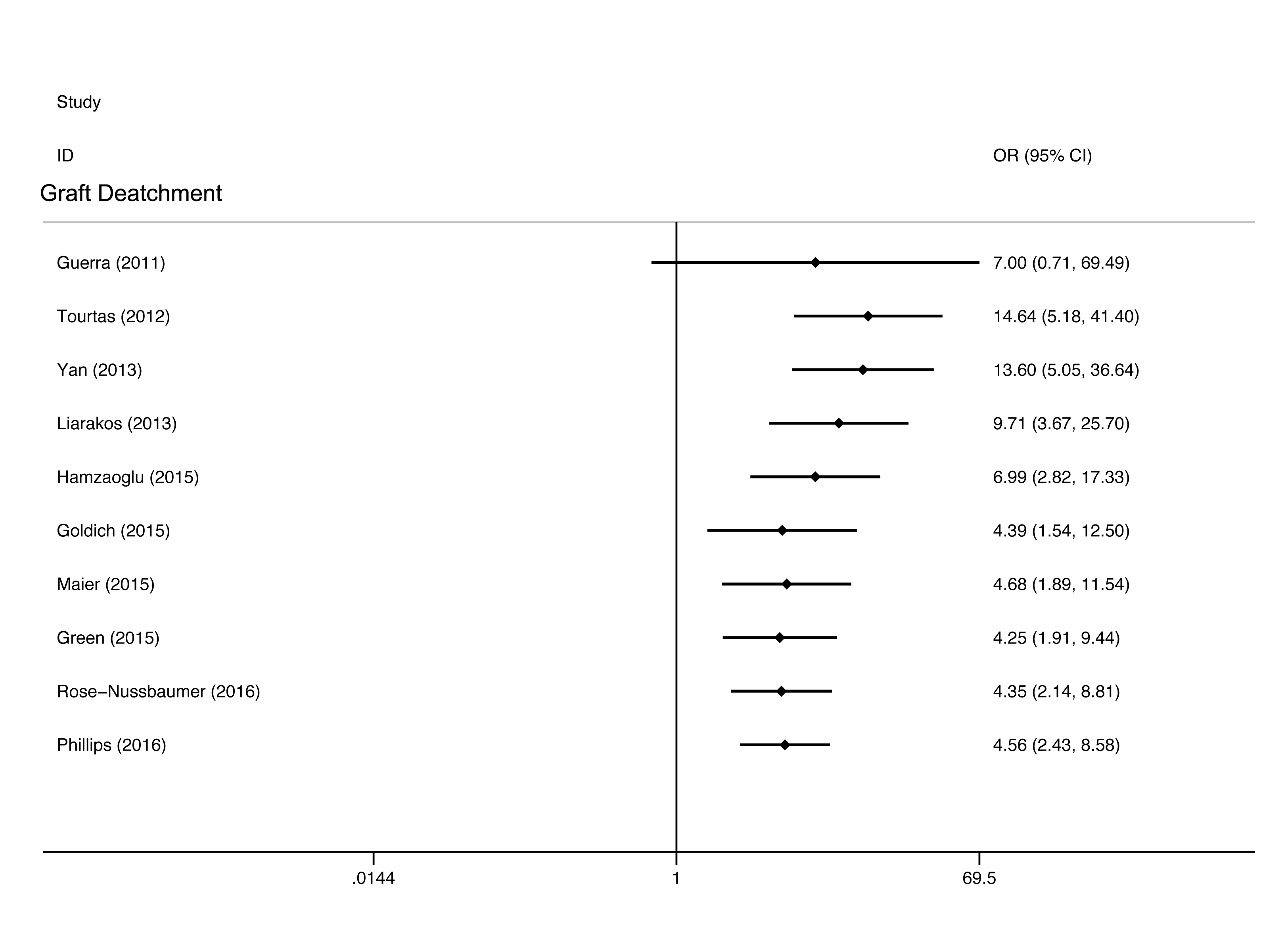

Supplement: S3 Fig — ES = effects estimates; CI = confidence interval. (TIF) [file pone.0182275.s006.tif]

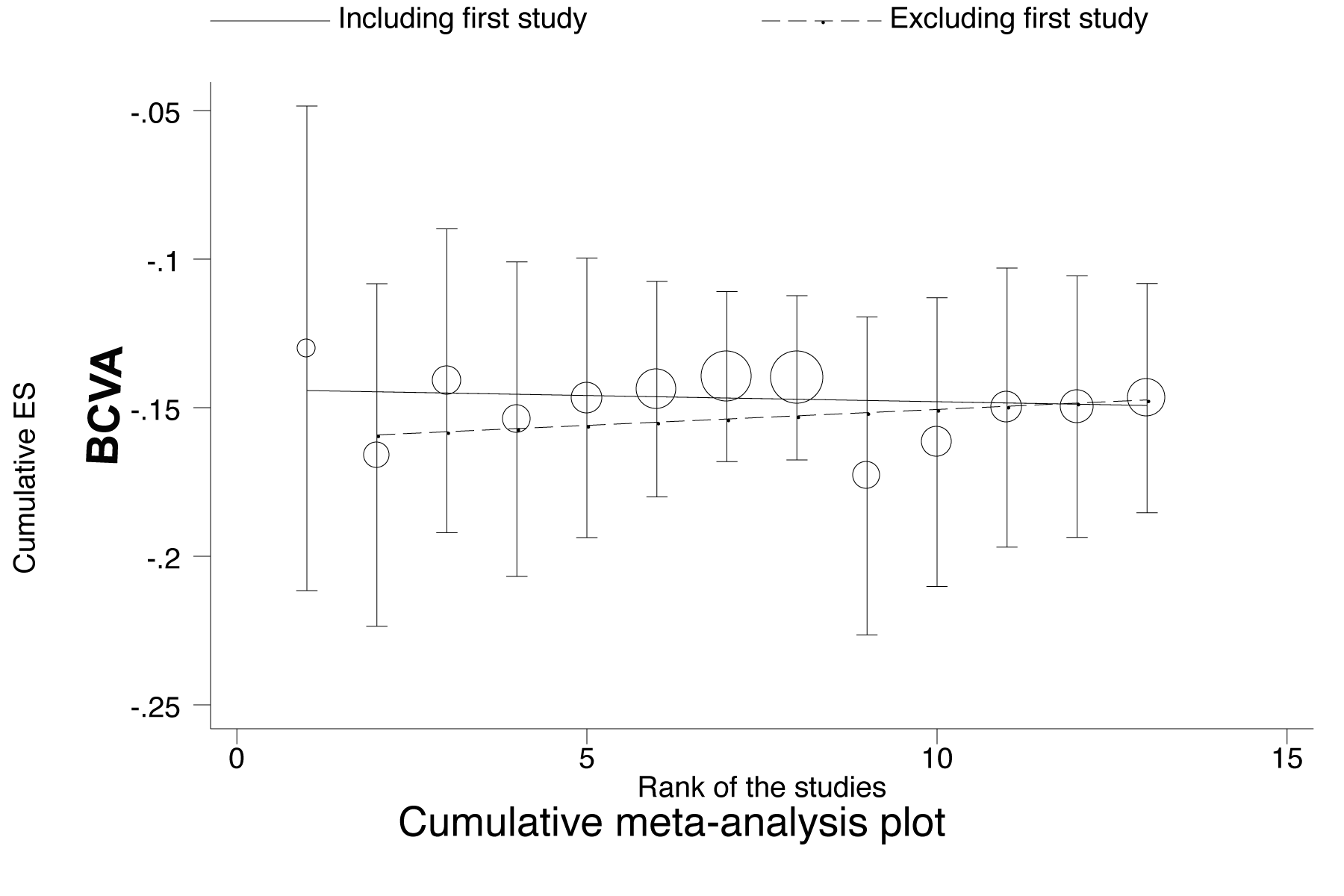

Supplement: S4 Fig — First vs Subsequent method: P = 0.690. GLS regression-based test: including all studies: Coef. = -0.00042, P = 0.612; excluding first studies: Coef. = 0.00107, P = 0.398. ES = effects estimates. (TIF) [file pone.0182275.s007.tif]

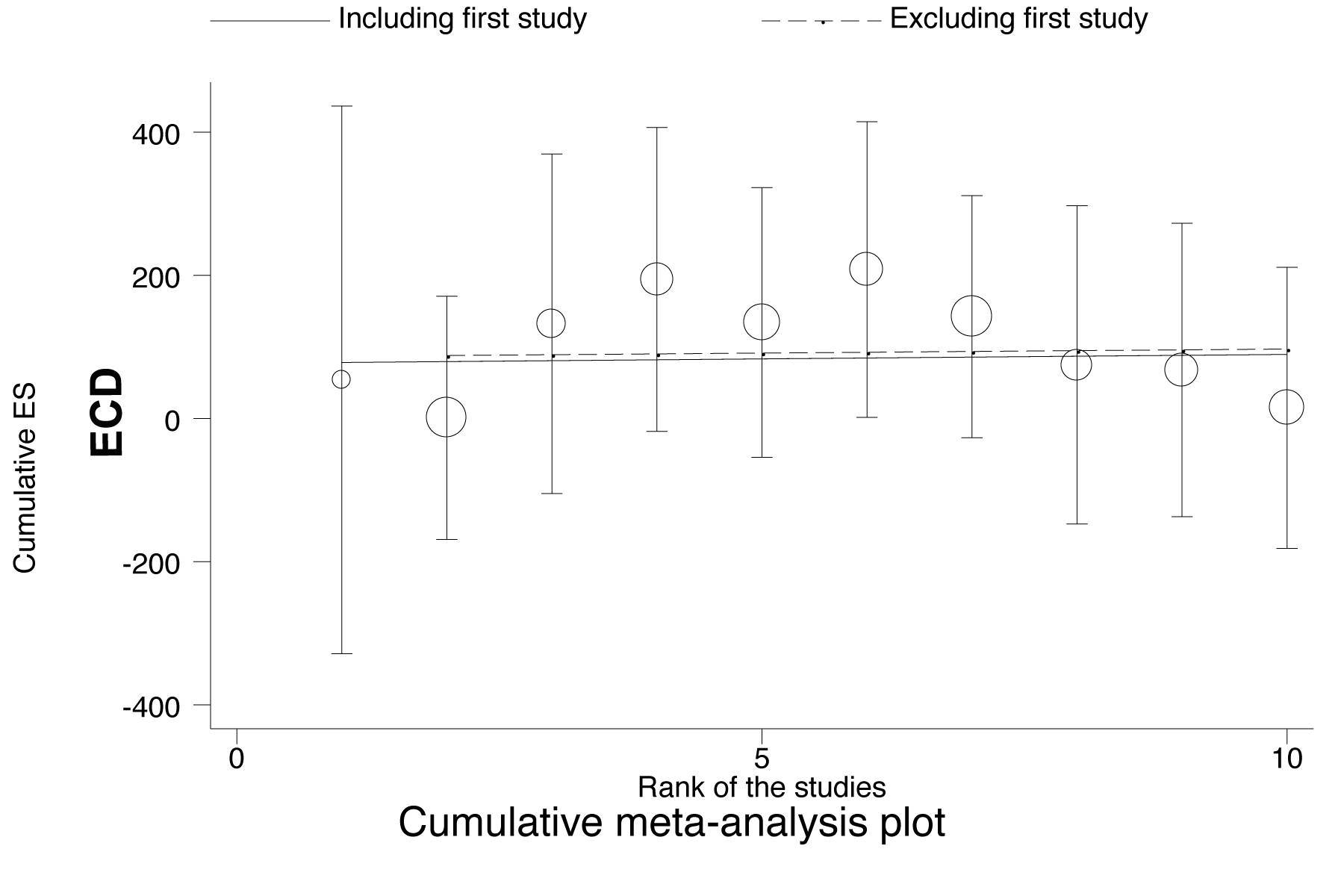

Supplement: S5 Fig — First vs Subsequent method: P = 0.850. GLS regression-based test: including all studies: Coef. = 1.249, P = 0.903; excluding first studies: Coef. = 1.136, P = 0.924. ES = effects estimates. (TIF) [file pone.0182275.s008.tif]

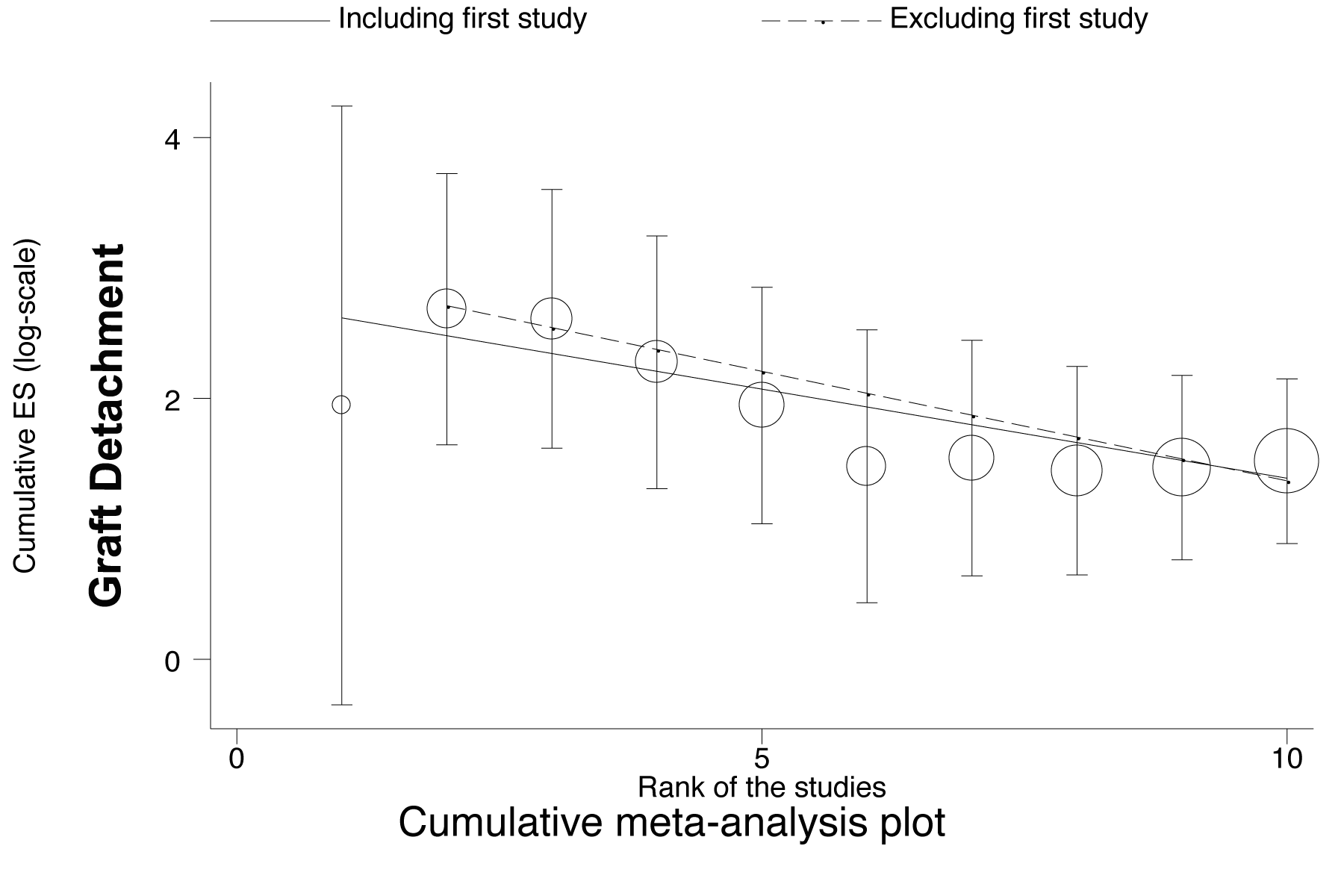

Supplement: S6 Fig — First vs Subsequent method: P = 0.686. GLS regression-based test: including all studies: Coef. = -0.137, P<0.001; excluding first studies: Coef. = -0.168, P<0.001. ES = effects estimates. (TIF) [file pone.0182275.s009.tif]
